# Updated Nematocyst Types in Tentacle of Venomous Box Jellyfish, *Chironex indrasaksajiae* _(Sucharitakul, 2017)_ and *Chiropsoides buitendijki* _(Horst, 1907)_ (Cnidaria, Cubozoa) in Thai Waters

**DOI:** 10.3390/toxins17010044

**Published:** 2025-01-17

**Authors:** Thippawan Yasanga, Klintean Wunnapuk, Rochana Phuackchantuck, Lakkana Thaikruea, Thunyaporn Achalawitkun, Purinat Rungraung, Sineenart Santidherakul

**Affiliations:** 1 Medical Science Research Equipment Center, Faculty of Medicine, Chiang Mai University, Chiang Mai 50200, Thailand; 2 Research Administration Section, Faculty of Medicine, Chiang Mai University, Chiang Mai 50200, Thailand; rochana.p@cmu.ac.th; 3 Department of Forensic Medicine, Faculty of Medicine, Chiang Mai University, Chiang Mai 50200, Thailand; klintean.w@cmu.ac.th; 4 Working Group on Maritime Service Plan System Development, Region 11, Royal Thai Ministry of Public Health, Surat Thani 84000, Thailand; lakkana.t@cmu.ac.th; 5 Chumphon Fisheries Provincial Office, Department of Fisheries, Chumphon 86000, Thailand; thunya-flower@hotmail.com; 6 Marine and Coastal Resources Research Center, The Upper Gulf of Thailand, Department of Marine and Coastal Resources, Samut Sakhon 74000, Thailand; purinat03fuse@gmail.com

**Keywords:** Cubozoa, box jellyfish, nematocyst, morphological, stinging organelles, pray capture

## Abstract

The multiple-tentacle box jellyfish, *Chironex indrasaksajiae* (Sucharitakul, 2017) and *Chiropsoides buitendijki* (Horst, 1907), are venomous species found in Thai waters. They are responsible for numerous envenomations through their stinging organelles, nematocysts. These specialized microscopic structures discharge venom, yet detailed knowledge of their types and morphology in these species remains limited. This study updates the characterization of nematocyst types and features in *C. indrasaksajiae* and *C. buitendijki* using light and scanning electron microscopy for detailed examination. Four distinct nematocyst types were identified: banana-shaped microbasic p-mastigophores, oval-shaped microbasic p-rhopaloids, sub-spherical microbasic p-rhopaloids, and rod-shaped isorhizas. In *C. indrasaksajiae*, banana-shaped microbasic p-mastigophores exhibited significant intraspecific variability, ranging from 30.26 µm to 102.56 µm in length and 6.42 µm to 17.01 µm in width. Conversely, *C. buitendijki* showed a narrower size range, 72.17 µm to 98.37 µm in length and 10.73 µm to 16.48 µm in width, based on multiple individuals. The size ranges for the other nematocyst types were consistent across both species. This study enhances the understanding of nematocyst morphology in these box jellyfish, providing a foundation for further research on venom delivery mechanisms and improved management of jellyfish envenomations in Thai waters.

## 1. Introduction

Box jellyfish (Cubozoa) are among the most venomous marine organisms, presenting substantial ecological and public health challenges. Within this group, species from the genera *Chironex* and *Chiropsoides* stand out for their potent venom, which poses a significant threat to coastal communities in tropical regions, particularly in Thailand [1,2,3,4]. Human encounters with these jellyfish are increasing, driven by expanding coastal populations and intensified marine activities. Envenomations caused by these species can range from mild dermal irritation to severe systemic effects, including cardiovascular collapse [5,6,7], and are associated with high rates of hospitalization and mortality in the Indo-Pacific region. Globally, over 150 million jellyfish stings are reported annually [8], disrupting vital coastal industries such as tourism, aquaculture, and fishing [9]. These impacts highlight the pressing need for deeper insights into the biology and venom mechanisms of these organisms, to improve sting management and mitigate their effects.

The venom delivery system of box jellyfish is centered on nematocysts—specialized stinging organelles that are unequaled in their complexity and efficiency. These organelles play a crucial role in prey capture and defense, highlighting their complicated functionality [10,11,12,13,14,15]. Upon mechanical or chemical stimulation, nematocysts deliver venom through everted tubules in milliseconds, making them one of the fastest known biological processes [12,16,17,18]. These structures are integral to prey capture and defense, and are highly variable across species, influencing venom potency and the severity of envenomation [19,20,21]. Nematocysts are critical for understanding species-specific venom delivery mechanisms and have recently gained attention as additional taxonomic markers in jellyfish, complementing their established role in other cnidarian orders, such as Actiniaria, for taxonomy [22]. They also provide valuable insights into the ecological adaptations of cnidarians. Variations in nematocyst morphology, including size, shape, and type, have been linked to environmental conditions, life stages, and dietary factors, underscoring their functional and ecological significance [11,23,24].

Despite extensive research on nematocysts across cnidarian classes, including Hydrozoa, Anthozoa, and Scyphozoa [25,26,27,28,29,30,31,32,33,34,35,36,37,38,39,40,41,42]. Gershwin (2006) [29] provides a detailed classification of jellyfish nematocysts, particularly in Cubozoa, defining key types such as p-mastigophores and p-rhopaloids. P-mastigophores are characterized by a V-shaped notch in the undischarged shaft, a feature that facilitates deep tissue penetration and efficient venom delivery. In contrast, p-rhopaloids exhibit a lobed structure upon discharge, reflecting adaptations suited for venom delivery at shallower depths or targeting specific tissue types. These morphological distinctions underscore the functional specialization of nematocysts and their ecological significance.

This classification not only aids in identifying jellyfish species but also highlights the relationship between nematocyst structure and function, providing valuable insights into their envenomation strategies. By integrating detailed morphological studies, such as those by Gershwin (2006) [29], with ecological and molecular data, we can further refine our understanding of jellyfish venom dynamics and their role in marine ecosystems.

However, comprehensive studies of nematocyst morphology in *Chironex indrasaksajiae* (Sucharitakul, 2017) [43] and *Chiropsoides buitendijki* (Horst, 1907) [44], two medically significant species in Thai waters [3,43,45,46], are notably lacking. Previous studies have documented the nematocyst morphology of *Chironex indrasaksajiae* and *Chiropsoides buitendijki*, offering foundational insights into their cnidom composition. Sucharitakul et al. (2017) [43] identified two nematocyst types in *Chironex indrasaksajiae*: oval-shaped trirhopaloids and cucumber-shaped microbasic p-mastigophores. Gershwin (2006a, 2006b) [29,44] described various nematocyst types in *Chiropsoides buitendijki*, including elongate club-shaped and large oval microbasic p-mastigophores, and football-shaped microbasic p-rhopaloids. This study expands on these works by updating the descriptions of nematocyst types, dimensions, and structural features observed in these species from Thai waters. This knowledge gap is particularly critical given the variation in nematocyst characteristics, which can assist in species identification and guide clinical management plans, even though the specific contributions of venom composition to envenomation outcomes remain to be fully clarified.

Box jellyfish stings in Thailand, particularly during peak tourist season, are a significant public health concern. *Chironex indrasaksajiae* and *Chiropsoides buitendijki* are the primary chirodropid species, with *Chiropsoides buitendijki* more frequently reported [46]. However, misidentifications between these species are common due to overlapping habitats and morphological similarities, highlighting the importance of cnidom-based identification. *Chironex indrasaksajiae* stings are highly venomous, causing severe pain, systemic effects, and fatalities. In contrast, *Chiropsoides buitendijki* stings result in mild symptoms and are managed symptomatically [46,47,48].

This study aims to address this gap by providing a comprehensive morphological characterization of nematocysts in *Chironex indrasaksajiae* and *Chiropsoides buitendijki*. Using advanced light and scanning electron microscopy techniques, we analyze the size, shape, and structural variability of nematocysts in these species. By elucidating their morphological traits, this research contributes to a deeper understanding of jellyfish venom systems, advances species-specific taxonomy, and informs practical applications and public safety measures in regions impacted by jellyfish stings.

## 2. Results

### 2.1. Morphological Characterization and Distribution of Nematocysts in the Tentacles of Chironex indrasaksajiae

**Figure 1:**
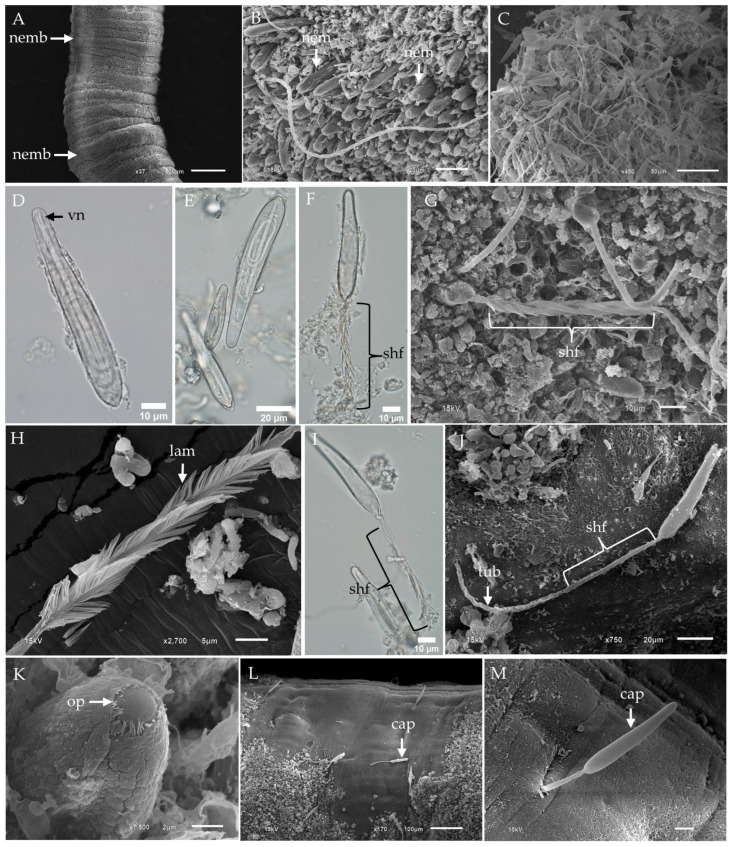
Light micrographs (LM) and scanning electron micrographs (SEM) of tentacles and banana-shaped microbasic p-mastigophore nematocysts from *Chironex indrasaksajiae*. (**A**) Low-magnification SEM of the fixed tentacle shows nematocyst bands (nemb). (**B**) Top-view SEM of the tentacle surface reveals nematocysts (nem) embedded within the tissue. (**C**) SEM of discharged nematocysts clustered on the tentacle surface highlights banana-shaped microbasic p-mastigophores as the dominant type. (**D**) LM of an undischarged banana-shaped microbasic p-mastigophore shows a distinct V-shaped notch (vn) at the distal end of the shaft. (**E**) LM of undischarged banana-shaped microbasic p-mastigophores demonstrates size variation. (**F**) LM of a fully discharged banana-shaped microbasic p-mastigophore reveals a right-handed helical spine shaft (shf). (**G**) SEM of the tentacle surface displays a discharged banana-shaped microbasic p-mastigophore. (**H**) Enlarged SEM of the right-handed helical spine shaft highlights well-spread lamellae (lam). (**I**) LM of an incompletely discharged banana-shaped microbasic p-mastigophore shows partially spread lamellae and spines visible on the shaft. (**J**) SEM of discharged banana-shaped microbasic p-mastigophores reveals an absence of visible spines on the shaft. (**K**) SEM top view of a banana-shaped microbasic p-mastigophore shows a circular-shaped operculum (op). (**L**,**M**) SEM images of the tentacle surface display a discharged p-mastigophore penetrating the tentacle’s tissue. **Abbreviations:** cap, capsule; lam, lamellae; nem, nematocyst; nemb, nematocyst band; op, operculum; shf, shaft; tub, tubule; vn, V-shaped notch.

The fixed tentacle of *Chironex indrasaksajiae* displayed numerous nematocyst bands, as visualized through scanning electron microscopy (SEM) (Figure 1A). At higher magnification, nematocysts were observed to be arranged in clusters on the outer surface of the tentacle (Figure 1B). Among the discharged nematocysts, banana-shaped microbasic p-mastigophores were the most frequently observed type (Figure 1C). Undischarged banana-shaped microbasic p-mastigophores exhibited elongated capsules, slightly broader at the apical end, with a coiled tubule and shaft inside. The shaft displayed a V-shaped notch at their distal end (Figure 1D). Isolated undischarged nematocysts of this type ranged in size from 30.26 µm to 102.56 µm in length (Figure 1E).

Fully discharged microbasic p-mastigophores were characterized by empty capsules and fully everted spined shafts, as observed in Figure 1F. SEM imaging revealed the shaft lamellae arranged in a helical pattern (Figure 1G,H). Partially discharged nematocysts displayed poorly spread lamellae (Figure 1I), while some lacked visible spines on the shaft (Figure 1J). At higher magnifications, the apical region of undischarged nematocysts exhibited a circular-shaped operculum (Figure 1K). Interestingly, discharged banana-shaped microbasic p-mastigophores were occasionally observed penetrating into their own tentacle tissue, potentially due to proximity during discharge (Figure 1L,M).

**Figure 2:**
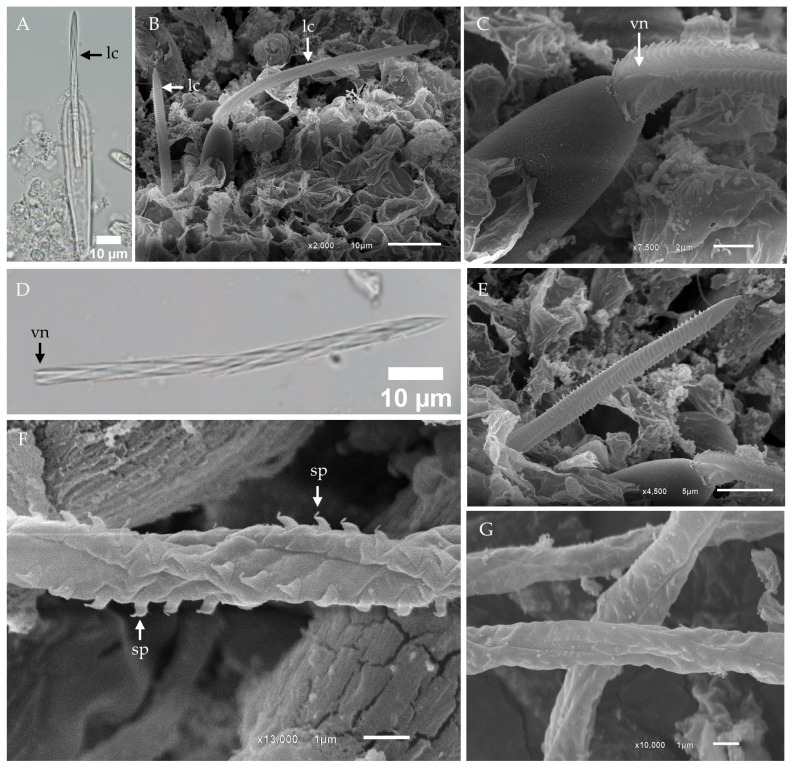
Light micrographs (LM) and scanning electron micrographs (SEM) of the lancet structure and tubule in banana-shaped microbasic p-mastigophores from *Chironex indrasaksajiae*. (**A**) LM of the initial discharge phase of banana-shaped microbasic p-mastigophores reveals the protruding lancet (lc) emerging from the capsule. (**B**) SEM shows the appearance of a long lancet on the tentacle surface, exhibiting a left-handed helical pattern. (**C**) SEM close-up of the V-shaped notch region of the lancet as it emerges from the capsule. (**D**) LM of a detached lancet with a prominent V-shaped notch at its distal end, observed to fall out following discharge. (**E**) SEM close-up of the tip of the lancet, highlighting its structural details. (**F**) SEM of the discharged tubule from a banana-shaped microbasic p-mastigophore shows arrow-shaped spines (sp) arranged in a helical pattern along its surface. (**G**) SEM of the tubule with spines partially enclosed within the membrane, indicating incomplete discharge. **Abbreviations:** lc, lancet; sp, spine; vn, V-shaped notch.

Light microscopy of isolated banana-shaped microbasic p-mastigophores captured the initial discharge phase, revealing partial eversion of the lancet—a spearhead-like tip structure—from the capsule (Figure 2A). During discharge, the compressed shaft was expelled as a dense projectile, subsequently expanding into an elongated cylinder with a distinct helical pattern. For consistency, we adopt the term “lancet” to describe the spearhead-like structure, as defined by Yanagihara et al. (2002) [41].

Scanning electron microscopy (SEM) further revealed the presence of lancets on the outer surface of the tentacle (Figure 2B). At higher magnification, the lancet exhibited a distinct V-shaped notch at its distal end (Figure 2C). In isolated samples, fully detached lancets were observed, providing additional morphological insights (Figure 2D). Closer inspection confirmed the lancet’s left-handed helical structure and its spearhead-like tip, further emphasizing its specialized design (Figure 2E).

Fully discharged banana-shaped microbasic p-mastigophores were composed of a capsule, a spine-covered shaft, and an everted tubule. The tubule surface displayed spines arranged in a distinct helical pattern, with each spine characterized by a broad, arrow-shaped base and a claw-like tip (Figure 2F). In instances of incomplete discharge, the tubule remained partially enclosed within the surrounding membrane, suggesting an intermediate stage of eversion (Figure 2G).

Other nematocyst types identified in the tentacles of *Chironex indrasaksajiae* include oval-shaped microbasic p-rhopaloids, sub-spherical microbasic p-rhopaloids, and rod-shaped isorhizas.

**Figure 3:**
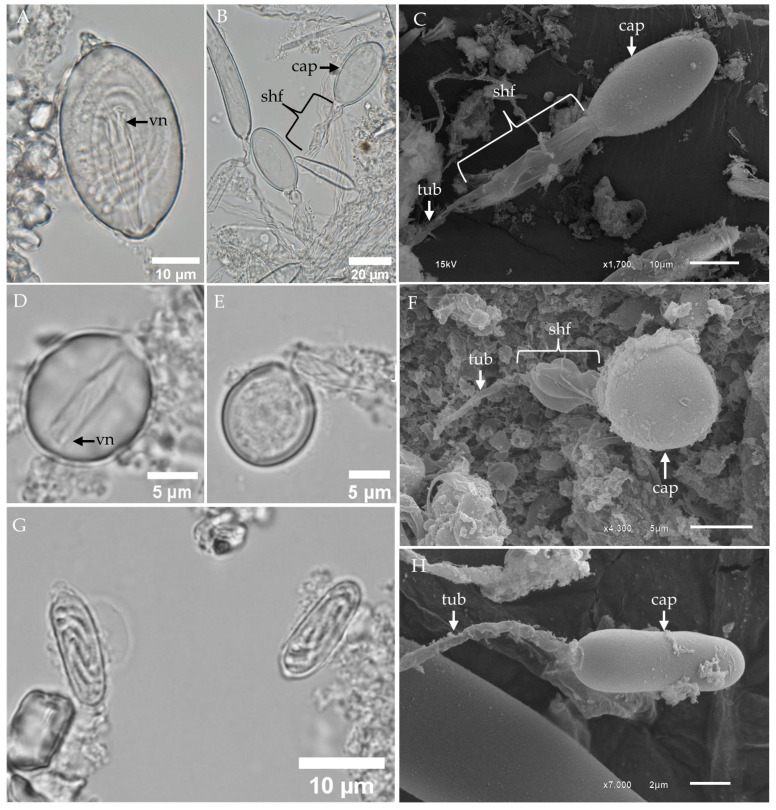
LM and SEM of the subdominant tentacular nematocysts of *Chironex indrasaksajiae*. (**A**) LM of undischarged oval-shaped microbasic p-rhopaloids shows the coiled tubule inside the capsule and a shaft with a V-shaped notch at the distal end. (**B**) LM and (**C**) SEM of discharged oval-shaped microbasic p-rhopaloids highlight the structural changes post-discharge. (**D**) LM of an undischarged small sub-spherical microbasic p-rhopaloid reveals the capsule (cap) and shaft. (**E**) LM and (**F**) SEM of discharged small sub-spherical microbasic p-rhopaloids show the structural details of the dilated shaft and everted tubule (tub). (**G**) LM of an undischarged rod-shaped isorhiza reveals the coiled tubule within the small, rod-shaped capsule. (**H**) SEM of discharged rod-shaped isorhizas highlights the smooth capsule wall and slender tubule. **Abbreviations:** cap, capsule; shf, shaft; tub, tubule; vn, V-shaped notch.

The undischarged oval-shaped microbasic p-rhopaloid displayed a distinct shaft with a V-shaped notch, surrounded by a coiled tubule within the capsule (Figure 3A). Upon discharge, the shaft length was approximately equal to the capsule length and exhibited distinct dilation (Figure 3B,C). Notably, spines on the shaft were not observed in this examination (Figure 3C).

Sub-spherical microbasic p-rhopaloids were identified in smaller numbers (Figure 3D–F). Light microscope revealed undischarged capsules with a shaft featuring a V-shaped notch (Figure 3D). During the initial discharge phase, the lancet everted from the capsule (Figure 3E). SEM analysis of discharged sub-spherical microbasic p-rhopaloids showed a distinctly dilated shaft, which also lacked spines (Figure 3F).

The rod-shaped isorhizas, the fourth type of nematocyst observed, were characterized by small, rod-shaped undischarged capsules containing a coiled tubule (Figure 3G). SEM analysis of discharged rod-shaped isorhizas revealed smooth capsule walls and slender tubules, with no visible shaft or spines (Figure 3H).

### 2.2. Morphological Characterization and Distribution of Nematocysts in the Tentacles of Chiropsoides buitendijki

**Figure 4:**
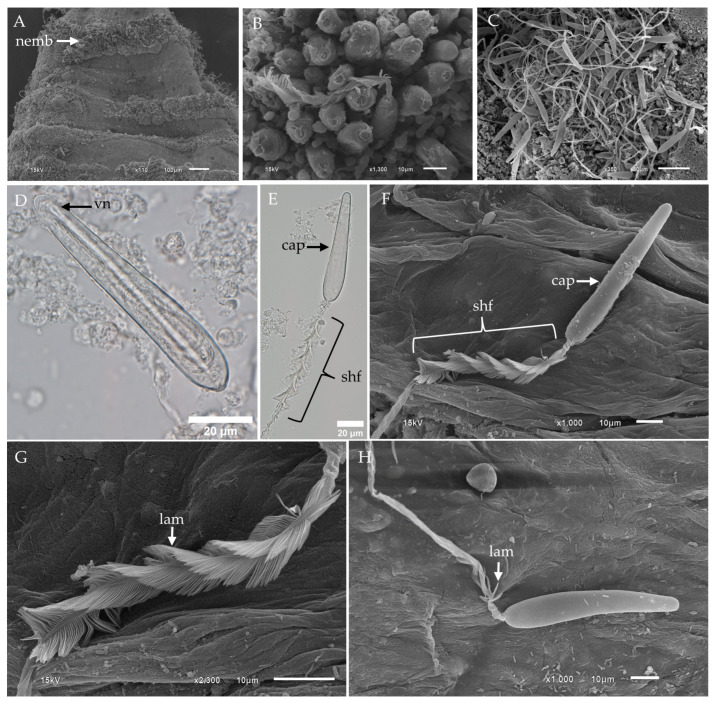
LM and SEM of tentacle and banana-shaped microbasic p-mastigophores of *Chiropsoides buitendijki*. (**A**) Low-magnification SEM of the tentacle reveals a nematocyst band with clusters of undischarged nematocysts. (**B**) High-magnification SEM top view of nematocysts on the tentacle surface shows embedded clusters. (**C**) SEM of discharged nematocysts on the surface of a fixed tentacle highlights the large population of banana-shaped microbasic p-mastigophores. (**D**) LM of isolated banana-shaped microbasic p-mastigophores shows a V-shaped notch at the distal end of the shaft. (**E**) LM of discharged banana-shaped microbasic p-mastigophores. (**F**) SEM of fully discharged banana-shaped microbasic p-mastigophores reveals a right-handed helical spine shaft. (**G**) SEM close-up of the spine shaft shows fully splayed lamellae. (**H**) SEM of incompletely discharged nematocysts shows lamellae that are not fully spread. **Abbreviations:** cap, capsule; lam, lamellae; nemb, nematocyst band; shf, shaft; tub, tubule; vn, V-shaped notch.

In this present study, the predominant nematocyst type identified in *Chiropsoides buitendijki* was the banana-shaped microbasic p-mastigophore. SEM analysis of the fixed tentacle revealed a distinct nematocyst band (Figure 4A), while close-up imaging of the outer surface showed clusters of undischarged nematocysts embedded together, with their apical parts exposed (Figure 4B). Clusters of discharged nematocysts on the tentacle surface were identified as banana-shaped microbasic p-mastigophores (Figure 4C).

Under light microscopy, the undischarged banana-shaped microbasic p-mastigophore exhibited an elongated capsule, broadest at the apical region, with a long shaft featuring a clearly visible V-shaped notch, surrounded by a coiled tubule (Figure 4D). The structure of the discharged nematocyst comprised an empty capsule, a spine-covered shaft, and a tubule (Figure 4E,F). SEM images of the fully released spine shaft showed well-spread lamellae arranged in a right-handed helical pattern (Figure 4G). In contrast, cases of incomplete shaft release revealed lamellae that remained tightly packed (Figure 4H).

**Figure 5:**
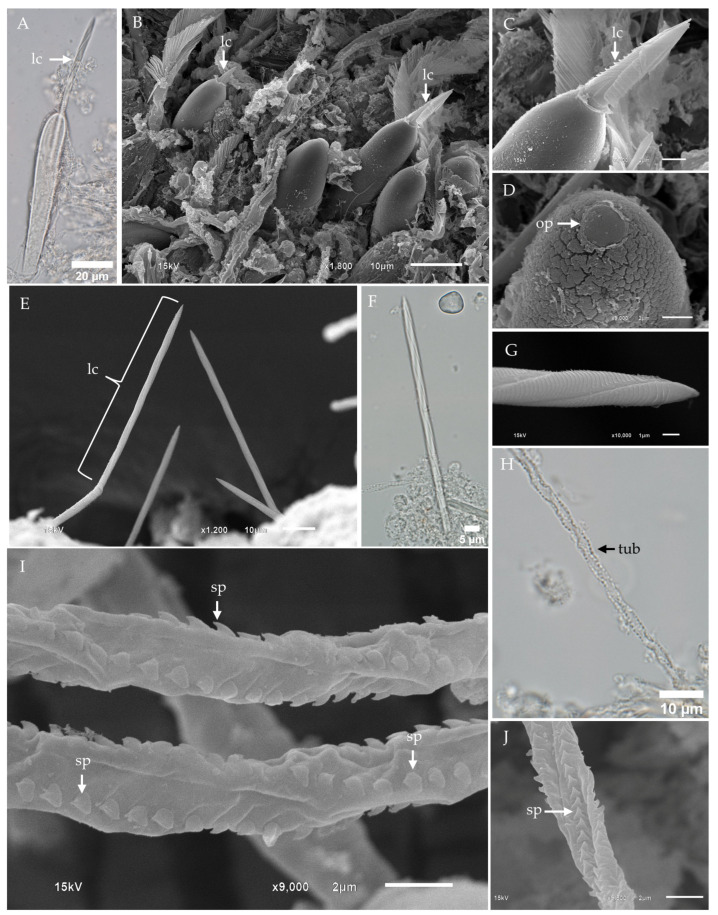
LM and SEM of lancet structure and tubule of banana-shaped microbasic p-mastigophores in *Chiropsoides buitendijki*. (**A**) LM showing the lancet initially emerging from the capsule. (**B**) SEM of the tentacle surface reveals banana-shaped microbasic p-mastigophores in the early stage of discharge, with the lancet becoming visible. (**C**) SEM close-up of the lancet tip as it begins to emerge from the capsule. (**D**) High-magnification SEM of the circular-shaped operculum of banana-shaped microbasic p-mastigophores. (**E**) SEM showing lancets observed on the tentacle surface. (**F**) LM of a free long lancet, noting that the lancet detaches from the shaft after full discharge. (**G**) High-magnification SEM of the lancet tip reveals a left-handed helical pattern. (**H**) LM of the tubule from a banana-shaped microbasic p-mastigophore, showing spines arranged in a helical pattern. (**I**,**J**) SEM of the tubule reveals numerous arrow-shaped spines. **Abbreviations:** lc, lancet; op, operculum; sp, spine; tub, tubule.

During the initial phase of banana-shaped microbasic p-mastigophore nematocyst discharge, the lancet partially everted from the capsule (Figure 5A). SEM images of the tentacle surface revealed lancet tips protruding from the nematocyst capsules (Figure 5B,C), while undischarged nematocysts retained a visible circular operculum (Figure 5D). Upon full discharge, the entire lancet length was observed on the tentacle surface (Figure 5E), consistent with the presence of free lancets in fixed, isolated samples (Figure 5F). High-magnification SEM of the lancet tip revealed a left-handed helical pattern (Figure 5G). Light microscopy of the discharged tubule showed spines arranged in a helical pattern along its surface (Figure 5H), while high-magnification SEM provided detailed views of the arrow-shaped spines with claw-like tips (Figure 5I,J).

Three subdominant nematocyst types were identified in the tentacles of *Chiropsoides buitendijki*: oval-shaped microbasic p-rhopaloids, sub-spherical microbasic p-rhopaloids, and rod-shaped isorhizas.

**Figure 6:**
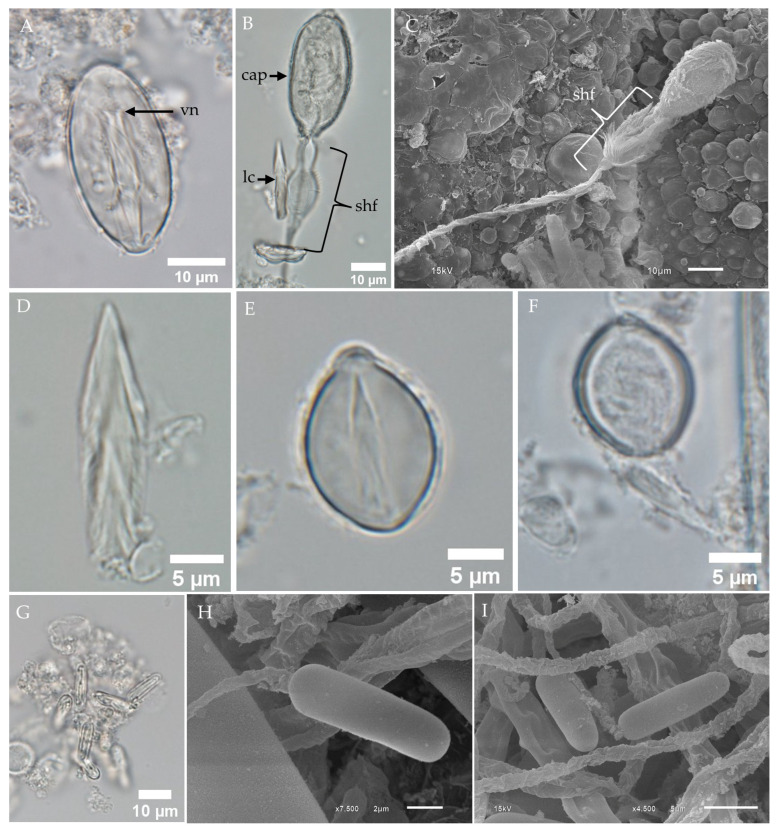
LM and SEM of the nondominant tentacular nematocysts of *Chiropsoides buitendijki*. (**A**) LM of undischarged oval-shaped microbasic p-rhopaloids. (**B**) LM of discharged oval-shaped microbasic p-rhopaloids showing a dilated shaft with an attached lancet (lc). (**C**) SEM of discharged oval-shaped microbasic p-rhopaloids. (**D**) LM of a free short lancet detached from the shaft of oval-shaped microbasic p-rhopaloids. (**E**) LM of undischarged small sub-spherical microbasic p-rhopaloid. (**F**) LM of discharged small sub-spherical microbasic p-rhopaloid. Note that small sub-spherical microbasic p-rhopaloids were not observed on the surface of fixed tentacles using SEM. (**G**) LM of undischarged rod-shaped isorhizas. (**H**,**I**) SEM of discharged rod-shaped isorhizas. **Abbreviations:** cap, capsule; lc, lancet; shf, shaft; vn, V-shaped notch.

LM of undischarged oval-shaped microbasic p-rhopaloids revealed the presence of a capsule, a shaft with a V-shaped notch, and a coiled tubule (Figure 6A). Discharged nematocysts observed under LM displayed an empty capsule, a dilated shaft, and a lancet attached near the shaft region (Figure 6B). SEM analysis of discharged nematocysts revealed a spine-covered shaft; however, the lancet was no longer visible (Figure 6C). Free lancets were observed in fixed, isolated samples (Figure 6D).

Sub-spherical microbasic p-rhopaloids were identified in small populations within the tentacles. Fixed, isolated samples examined under LM revealed both undischarged (Figure 6E) and discharged (Figure 6F) capsules.

Rod-shaped isorhizas were observed using both LM (Figure 6G) and SEM (Figure 6H,I). Undischarged rod-shaped isorhizas displayed coiled tubules within the capsules (Figure 6G). SEM images of discharged rod-shaped isorhizas revealed capsules with released tubules that lacked spines (Figure 6H,I).

### 2.3. Comparison of Nematocyst Sizes Between Chironex indrasaksajiae and Chiropsoides buitendijki

This study identified four nematocyst types in the tentacles of *Chironex indrasaksajiae* and *Chiropsoides buitendijki*: banana-shaped microbasic p-mastigophores, oval-shaped microbasic p-rhopaloids, sub-spherical microbasic p-rhopaloids, and rod-shaped isorhizas. While all types were present in both species, significant size variation was observed only in the banana-shaped microbasic p-mastigophores (*p* < 0.01; Table 1). In *Chironex indrasaksajiae*, these nematocysts exhibited a wider size range (30.26–102.56 µm in length, mean = 58.05 ± 15.46 µm; 6.42–17.01 µm in width, mean = 9.74 ± 1.88 µm) compared to *Chiropsoides buitendijki* (72.17–98.37 µm in length, mean = 85.22 ± 4.21 µm; 10.73–18.48 µm in width, mean = 13.84 ± 1.48 µm).

Measurements of the other nematocyst types showed no significant differences between the two species. In *Chironex indrasaksajiae*, oval-shaped microbasic p-rhopaloids ranged from 30.27 to 43.50 µm in length (mean = 36.79 ± 3.22 µm) and 15.89–24.66 µm in width (mean = 19.85 ± 2.17 µm). Sub-spherical microbasic p-rhopaloids measured 11.02–15.50 µm in length (mean = 13.28 ± 0.95 µm) and 8.85–14.51 µm in width (mean = 10.84 ± 1.10 µm). Rod-shaped isorhizas ranged from 10.70 to 14.95 µm in length (mean = 12.93 ± 0.93 µm) and 4.01–7.78 µm in width (mean = 4.80 ± 0.57 µm). Similarly, in *Chiropsoides buitendijki*, oval-shaped microbasic p-rhopaloids measured 30.71–41.10 µm in length (mean = 36.03 ± 1.99 µm) and 15.25–23.72 µm in width (mean = 18.23 ± 2.27 µm). Sub-spherical microbasic p-rhopaloids ranged from 13.92 to 19.50 µm in length (mean = 15.70 ± 0.95 µm) and 10.03–14.75 µm in width (mean = 11.93 ± 0.84 µm). Rod-shaped isorhizas were 12.25–16.06 µm in length (mean = 14.13 ± 0.83 µm) and 3.37–5.14 µm in width (mean = 3.97 ± 0.30 µm) (Table 1).

## 3. Discussion

This study identified four nematocyst types in the tentacles of *Chironex indrasaksajiae* and *Chiropsoides buitendijki*: banana-shaped microbasic p-mastigophores, oval-shaped microbasic p-rhopaloids, sub-spherical microbasic p-rhopaloids, and rod-shaped isorhizas. While all types were present in both species, the banana-shaped microbasic p-mastigophores presented significant intraspecific variability, particularly in *Chironex indrasaksajiae.*

The dimensions of banana-shaped microbasic p-mastigophores in *Chironex indrasaksajiae* ranged from 30.26 to 102.56 µm in length, as measured from a single specimen. While this range may suggest developmental or physiological influences, it is important to note that the data are limited to one individual and may not capture the full variability within the species. Further studies involving multiple specimens are necessary to determine whether this range represents intraspecific variation or includes multiple nematocyst types. In contrast, *Chiropsoides buitendijki* exhibited a narrower range of 72.17–98.37 µm, consistent with reduced variability observed across multiple individuals. However, the broader size variability in banana-shaped microbasic p-mastigophores of *Chironex indrasaksajiae* may be influenced by factors such as developmental stage, environmental conditions, genetic determinants of nematocyst formation, or differences in predatory behaviors, highlighting species-specific adaptations to ecological niches. These findings align with prior studies on *Chironex fleckeri* [29,49], where significant size variation was attributed to individual and environmental factors.

On the other hand, the sizes of oval-shaped microbasic p-rhopaloids, sub-spherical microbasic p-rhopaloids, and rod-shaped isorhizas remained consistent between the two species, indicating that these nematocyst types are less affected by such variability. The detailed imaging of banana-shaped microbasic p-mastigophores was prioritized due to their significant size variability and functional importance in deep tissue penetration and venom delivery. Conversely, oval-shaped microbasic p-rhopaloids, sub-spherical microbasic p-rhopaloids, and rod-shaped isorhizas were observed in much smaller quantities, making their detailed imaging more challenging. Their low abundance and smaller dimensions required more extensive sample preparation and imaging efforts, which were beyond the scope of the current study. These constraints highlight the need for future research focused on the less abundant nematocyst types to provide a more comprehensive understanding of their morphology and functional roles.

This study expands the known diversity of nematocysts in *Chiropsoides buitendijki*, adding sub-spherical microbasic p-rhopaloids and rod-shaped isorhizas to the three types previously described by Gershwin (2006) [29]. Differences in nematocyst diversity could stem from environmental factors, sampling methods, or specimen sources, warranting further investigation into these influences. Sucharitakul et al. (2017) [43] reported oval-shaped trirhopaloids and cucumber-shaped microbasic p-mastigophores *in Chironex indrasaksajiae*. Our findings extend this work by documenting banana-shaped microbasic p-mastigophores, characterized by their elongated shafts and prominent spines, which may reflect developmental or morphological variations. Additionally, we have documented sub-spherical microbasic p-rhopaloids and rod-shaped isorhizas, further enhancing the understanding of nematocyst diversity in this species.

The significant size variability of banana-shaped microbasic p-mastigophores in *Chironex indrasaksajiae* compared to *Chiropsoides buitendijki* may be attributed to several factors. This variability could reflect differences in developmental stages, as maturing nematocysts often exhibit size variation. Additionally, environmental conditions, such as water temperature, salinity, and prey availability, as well as genetic factors influencing nematocyst formation, may contribute to the broader intraspecific variation observed in *Chironex indrasaksajiae*. These findings underscore the importance of further research to understand how such variability may relate to ecological and functional adaptations in different cubozoan species.

While this study did not specifically investigate these ecological correlations, the findings highlight the potential for future research to explore how nematocyst morphology aligns with species-specific behaviors and environmental adaptations.

The structural differences among the nematocyst types observed in this study highlight their specialized functional roles during envenomation. Banana-shaped microbasic p-mastigophores, with their elongated shafts and prominent spines, are well-suited for deep tissue penetration and efficient venom delivery, consistent with previous findings linking open-tubule nematocysts to penetrant functions [50]. In contrast, the consistent morphology of oval-shaped and sub-spherical microbasic p-rhopaloids suggests roles in delivering venom at shallower tissue depths or immobilizing prey, while rod-shaped isorhizas may function in adhesion or substrate attachment. These functional distinctions align with ecological and evolutionary factors, as nematocyst morphology and size are known to vary along with environmental conditions, developmental stages, and prey types [51]. Additionally, the venom delivery mechanism via spines on the everted tubule surface underscores structural adaptations enhancing venom efficacy [52]. These findings emphasize the ecological, physiological, and behavioral significance of nematocyst diversity in cnidarians.

Although our study did not focus on the detailed mechanics of venom discharge, the observed discharge patterns of banana-shaped microbasic p-mastigophores, including tubule eversion and lancet deployment, align with the three-phase discharge model proposed in recent studies [53]. These findings reinforce the conserved nature of this mechanism across cubozoans, emphasizing the need for future research to investigate species-specific adaptations in venom delivery. SEM images revealed the initial emergence of the lancet, its helical structure, and sub-sequent tubule eversion, suggesting a conserved discharge process among cubozoans. Although the biological significance of the left-handed and right-handed helical patterns in nematocyst shafts remains speculative, studies by Beckmann and Özbek (2012) [12] propose that these helical patterns may enhance mechanical stability and discharge dynamics, potentially optimizing the efficiency of penetration and venom delivery.

The circular operculum observed in *Chironex indrasaksajiae* and *Chiropsoides buitendijki* mirrors those of other cubozoan species, including *Chironex fleckeri* [49], reinforcing its role in facilitating efficient tubule eversion and venom delivery. In contrast, variations in operculum shape, such as triangular structures in *Carybdea alata* [41], highlight potential ecological adaptations.

The nematocyst features of *Chironex indrasaksajiae* and *Chiropsoides buitendijki* exhibit both similarities and distinctions compared to other venomous jellyfish species, such as *Chironex fleckeri*. Shared nematocyst types, including banana-shaped microbasic p-mastigophores, heterotrichous euryteles, and isorhizas, underscore their common functional roles in venom delivery and prey capture. Significant size variability in the banana-shaped microbasic p-mastigophores of *Chironex indrasaksajiae*, compared to *Chironex fleckeri*, likely reflects species-specific adaptations to ecological niches and predatory behaviors. These findings emphasize the importance of nematocyst diversity in shaping the ecological and functional strategies of cubozoans and align with prior studies on *Chironex fleckeri* [29,49], which reported significant size variations influenced by individual and environmental factors. Moreover, the banana-shaped microbasic p-mastigophores observed in this study exhibit morphological similarities to those in Hydrozoan species, such as *Apolemia* sp. [42] and *Physalia physalis* [40], particularly in their spined tubule structure. These similarities suggest evolutionary convergence or shared functional adaptations in spined tubule and lancet morphology, potentially enhancing nematocyst deployment and prey capture efficiency.

In contrast, single-tentacle box jellyfish such as *Gershwinia thailandensis* and *Morbakka* sp. from Thai waters exhibit distinct nematocyst types, including club-shaped microbasic p-mastigophores (Type 4), oval isorhizas, and oval microbasic p-rhopaloids, as documented in our previous study [54]. Among these, only the oval microbasic p-rhopaloids overlap with those in multi-tentacle box jellyfish. These differences highlight the species-specific ecological and functional adaptations present within the diverse cubozoan lineage.

SEM observations revealed that banana-shaped microbasic p-mastigophores can penetrate their own tentacle tissue, indicating a random discharge mechanism triggered by external stimuli, such as mechanical or chemical interactions. Similar random discharge has been documented in other cnidarians, such as Hydra [55], and is thought to serve defensive or regulatory purposes. In *Chironex indrasaksajiae* and *Chiropsoides buitendijki*, this phenomenon may represent an adaptive response to environmental pressures or a strategy to optimize venom deployment in crowded nematocyst batteries. The everted tubule, as described in Hydra [10], functions like a syringe, delivering venom with precision. These findings highlight the clinical relevance of understanding nematocyst variability and discharge mechanisms, which could inform strategies for managing envenomation incidents.

Identification of nematocyst types has been shown to aid in correlating clinical symptoms with jellyfish stings [56], underscoring the potential role of species-specific identification, based on nematocyst analysis, in improving sting management and informing treatment strategies. Our findings on the nematocyst diversity and functional specialization in *Chironex indrasaksajiae* and *Chiropsoides buitendijki* have significant implications for advancing the medical management of jellyfish envenomations. Understanding the structural and functional differences among nematocyst types could facilitate more accurate assessments of envenomation severity and support the development of species-specific first-aid protocols and antivenoms. Additionally, the elucidation of venom delivery mechanisms in this study provides a foundation for venom-based therapeutic research, particularly in identifying bioactive compounds with potential applications in pain management, anti-inflammatory treatments, and oncology.

The clinical variability of jellyfish stings, which range from localized reactions to systemic life-threatening events, highlights the importance of correlating nematocyst morphology and venom delivery mechanisms with clinical outcomes. Identifying specific nematocyst types, as demonstrated here, may improve diagnostic accuracy and enable tailored therapeutic interventions based on species-specific envenomation profiles. These insights underscore the need for continued research into nematocyst morphology and venom composition to bridge ecological findings with advancements in medical and biotechnological fields, as previously suggested by Lakkis et al. (2015) [57].

Building on our findings, future research will focus on the relationship between nematocyst morphology and venom composition, particularly the role of structural differences among nematocyst types in venom delivery and clinical outcomes of envenomation. Specifically, analyzing the biochemical properties of venom associated with banana-shaped microbasic p-mastigophores and microbasic p-rhopaloids could elucidate their functional roles in prey immobilization, tissue penetration, and toxicity. Understanding these mechanisms may enhance our ability to correlate nematocyst features with species-specific envenomation profiles, aiding in the development of targeted first-aid protocols and therapeutic strategies. Additionally, investigating the influence of environmental and physiological factors on nematocyst development and venom composition could provide deeper insights into species-specific adaptations. These studies will further bridge ecological findings with medical and biotechnological advancements, reinforcing the importance of nematocyst research in both ecological and applied contexts.

## 4. Conclusions

This study investigated the nematocyst types and characteristics in the multiple-tentacle box jellyfish *Chironex indrasaksajiae* and *Chiropsoides buitendijki*. Four nematocyst types were identified: banana-shaped microbasic p-mastigophores, oval-shaped microbasic p-rhopaloids, sub-spherical microbasic p-rhopaloids, and rod-shaped isorhizas. *Chironex indrasaksajiae* exhibited greater size variability in banana-shaped microbasic p-mastigophores compared to *Chiropsoides buitendijki*, likely due to differences in developmental stages, environmental factors, or genetic influences. These findings provide a basis for future studies on nematocyst mechanisms, venom composition, and sting management.

Using SEM for nematocyst characterization presented challenges, including preserving structural integrity during fixation and preparation, which sometimes led to distortion or incomplete discharge. The low abundance of certain nematocyst types on fixed tentacle samples further complicated the acquisition of representative images. These limitations, along with constraints in specimen availability and preservation, necessitated multiple attempts to capture sufficient data, and restricted our analysis to morphological and structural observations.

Quantitative analyses were limited by sample availability and preservation constraints, as this study focused on morphological and structural observations. Future research should expand specimen collection and standardize methods to clarify the drivers of nematocyst size variability and its biological significance.

## 5. Materials and Methods

### 5.1. Specimen Collection

Specimen collection followed methods adapted from Thaikruea and Santidherakul (2018) [58]. Box jellyfish specimens of *Chironex indrasaksajiae* and *Chiropsoides buitendijki* were collected from beaches in the Lower Gulf of Thailand with reported stinging incidents, including Tak Bai (Narathiwat Province), Sakom Beach (Songkhla Province), and Chaloklum Beach (Koh PhaNgan, Surat Thani Province). Tentacle samples were excised from the tip to the middle region, depending on the availability of intact structures, following modified procedures from Yanagihara et al. (2002) [41].

Specimen identification was performed by trained staff at the Marine and Coastal Resources Research Center, Department of Marine and Coastal Resources, Thailand. Whole-body samples were preserved for future reference following tentacle excision. Tentacle segments (1–2 cm) were immediately placed in distilled water for 1–2 min to induce nematocyst discharge and then fixed in 2.5% glutaraldehyde (Electron Microscopy Sciences, Hatfield, PA, USA) in 0.1 M PBS (pH 7.4) and stored at 4 °C.

Fixed tentacle samples were rinsed with PBS and prepared for scanning electron microscopy (SEM). The remaining fixative solution was centrifuged at 3000× *g* for 2 min to isolate nematocysts, which were rinsed with double-distilled water and processed for further analysis.

### 5.2. Analyses and Classification of Isolated Nematocysts by Light Microscopy

For nematocyst analysis, isolated nematocysts were suspended in a small amount of double-distilled water (DDW). The suspension was dropped directly onto a slide and examined using a BX63 Olympus microscope (Tokyo, Japan). The length and width of undischarged nematocysts were measured as direct lines from the posterior to the anterior ends of the capsule and perpendicularly across the widest point of the capsule, excluding curvature. Morphological features of both discharged and undischarged nematocysts were examined using a combination of light microscopy (BX63, Olympus microscope, Tokyo, Japan) and scanning electron microscopy (SEM, JSM 6610LV, JEOL, Tokyo, Japan). Nematocyst identification in this research follows the nomenclature and classification scheme of Gershwin (2006) [29].

1. Banana-shaped microbasic p-mastigophores
  This nematocyst type is characterized by an elongated capsule, widest at the apical region and tapering toward the distal end. Upon discharge, the shaft length is shorter than the capsule, with a shaft-to-capsule length ratio of less than 1.5. The undischarged shaft features a distinctive V-shaped notch at its base.
2. Oval-shaped microbasic p-rhopaloids
  These nematocysts have an oval-shaped capsule. The discharged shaft length is shorter than the capsule length, maintaining a shaft-to-capsule length ratio of less than 1.5. The undischarged shaft has a V-shaped notch at its base, while the discharged shaft displays prominent swellings.
3. Small sub-spherical microbasic p-rhopaloids
  This type features a small, sub-spherical capsule. Similarly to oval-shaped p-rhopaloids, the discharged shaft is shorter than the capsule length, with a shaft-to-capsule length ratio of less than 1.5. The undischarged shaft has a V-shaped notch at its base, and the discharged shaft exhibits prominent swellings.
4. Rod-shaped isorhizas
  Rod-shaped isorhizas are defined by their rod-shaped capsules, lacking a well-defined shaft. The discharged tubule maintains a uniform diameter or tapers slightly toward the distal end.

### 5.3. Scanning Electron Microscopy

Samples were fixed in 2.5% glutaraldehyde (Electron Microscopy Sciences, Hatfield, PA, USA) in 0.1 M PBS at 4 °C for at least 1 h, followed by post-fixation in 1% osmium tetroxide (Electron Microscopy Sciences, Hatfield, PA, USA) in double-distilled water for 1 h. They were then dehydrated in a graded ethanol series (50%, 70%, 85%, 95%, and 100%) and dried using a Quorum K850 Critical Point Dryer (Quorum Technologies, East Sussex, UK). The dehydrated samples were mounted on aluminum stubs and sputter-coated with gold using a Quorum Q150R S coater (Quorum Technologies, East Sussex, UK). All samples were observed and imaged using a scanning electron microscope (JSM 6610LV, JEOL, Tokyo, Japan).

### 5.4. Statistical Analysis

Due to limitations in sample collection, nematocyst measurements for *Chironex indrasaksajiae* were obtained from a single individual, whereas measurements for *Chiropsoides buitendijki* were derived from multiple individuals, providing a broader representation of variability across specimens. This approach reflects individual variability for *Chironex indrasaksajiae* and population-level data for *Chiropsoides buitendijki*.

The length and width of undischarged nematocyst capsules were measured from light microscopy images. The normality of the data was evaluated using the Shapiro–Wilk and Kolmogorov–Smirnov tests, as described by Acuña et al. (2004) [59] and Garese et al. (2016) [36]. Non-normal distributions in several groups necessitated the use of the Kruskal–Wallis test, followed by Dunn’s test for multiple comparisons. Data are presented as ranges (minimum–maximum) and mean ± standard deviations (SD). Statistical significance was set at *p* < 0.01. All analyses were performed using IBM SPSS software version 22.0 for Windows (IBM Corp., 2013, Armonk, NY, USA).

## Figures and Tables

**Table 1:** The capsule sizes of undischarged nematocyst types in *Chironex indrasaksajiae* and *Chiropsoides buitendijki*.

Species		*Chironex indrasaksajiae*		*Chiropsoides buitendijki*	
Type of Nematocyst		Capsule Length (µm)	Capsule Wide (µm)	Capsule Length (µm)	Capsule Wide (µm)
Banana-shaped microbasic p-mastigophores	Range (Min–Max)	30.26–102.56	6.42–17.01	72.17–98.37	10.73–18.48
	Mean ± SD	58.05 ± 15.46 ^ab^	9.74 ± 1.88 ^cd^	85.22 ± 4.21 ^ab^	13.84 ± 1.48 ^cd^
	(n)	(n = 323)		(n = 206)	
Oval-shaped microbasic p-rhopaloids	Range (Min–Max)	30.27–43.50	15.89–24.66	30.71–41.10	15.25–23.72
	Mean ± SD	36.79 ± 3.22	19.85 ± 2.17	36.03 ± 1.99	18.23 ± 2.27
	(n)	(n =42)		(n = 83)	
Small sub-spherical microbasic p-rhopaloids	Range (Min–Max)	11.02–15.50	8.85–14.51	13.92–19.50	10.03–14.75
	Mean ± SD	13.28 ± 0.95	10.84 ± 1.10	15.70 ± 0.95	11.93 ± 0.84
	(n)	(n = 37)		(n = 67)	
Rod-shaped isorhizas	Range (Min–Max)	10.70–14.95	4.01–7.78	12.25–16.06	3.37–5.14
	Mean ± SD	12.93 ± 0.93	4.80 ± 0.57	14.13 ± 0.83	3.97 ± 0.30
	(n)	(n = 72)		(n = 121)	

Note: The dimensions of nematocysts for *Chironex indrasaksajiae* are based on measurements from a single jellyfish specimen (N = 1), whereas measurements for *Chiropsoides. buitendijki* are based on multiple specimens (N = 2). Statistical differences in the dimensions of banana-shaped microbasic p-mastigophores between *Chironex indrasaksajiae* and *Chiropsoides buitendijki* are denoted by different letters (a–d), with significance at *p* < 0.01.

## Data Availability

The original contributions presented in this study are included in this article. Further inquiries can be directed to the corresponding authors.
